# Etiology and Anatomical Location of Uveitis—Prognostic Factors for Disease Course and Laterality

**DOI:** 10.3390/life15060882

**Published:** 2025-05-30

**Authors:** Vesela Todorova Mitkova-Hristova, Marin Anguelov Atanassov

**Affiliations:** Department of Ophthalmology, Faculty of Medicine, Medical University of Plovdiv, 4002 Plovdiv, Bulgaria; marin.atanassov@mu-plovdiv.bg

**Keywords:** uveitis, epidemiology, disease course, anatomical location, *HLA-B27*-associated uveitis

## Abstract

Background: Uveitis encompasses a broad group of diseases with infectious and non-infectious etiologies, potentially leading to permanent and irreversible visual impairment. This study aimed to determine whether the etiology and anatomical location of uveitis influence the course and laterality of the disease. Methods: This retrospective observational study included patients with uveitis treated at the University Eye Clinic of “St. George” University Hospital in Plovdiv, Bulgaria, between January 2011 and December 2023. All participants underwent a comprehensive eye examination and minimal laboratory screening, with additional tests and specialist consultations performed when necessary. Uveitis cases were categorized into anterior, intermediate, posterior, and panuveitis according to anatomical location; unilateral or bilateral according to laterality; and acute, chronic, or recurrent based on disease course. Results: The study included 606 patients aged between 3 and 87 years. The etiology and anatomical location of uveitis were significantly associated with the course and laterality of the disease (*p* < 0.001). Anterior and posterior uveitis were mostly unilateral, with a defined cause and characterized by acute or recurring progression. Intermediate uveitis was mostly idiopathic and chronic, while panuveitis had a more uniform distribution regarding disease progression. Among cases with a determined etiology, *HLA-B27*-associated uveitis was the most common (32.5%), characterized by sequential involvement of both eyes and a recurrent course. Viral and toxoplasmic uveitis were more frequently unilateral. Conclusions: Our findings indicate that the etiology and anatomical location of uveitis can serve as prognostic factors for contralateral eye involvement and the progression of the inflammatory process. We found that anterior and posterior uveitis were predominantly unilateral and typically presented with an acute or recurrent course, whereas intermediate uveitis and panuveitis were more commonly chronic. In men, uveitis more often had an acute onset, while in women, it tended to follow a chronic course.

## 1. Introduction

Uveitis encompasses a broad group of diseases with infectious and non-infectious etiologies. In many cases, the inflammatory process involves not only the uveal tract but also adjacent structures, including the retina, optic nerve, and vitreous body, and can lead to permanent and irreversible visual impairment. Related research has shown that demographic and personal factors can influence the incidence of the disease [1,2,3].

Uveitis primarily affects the working-age population, leading to numerous social and economic challenges [4]. Additionally, increasing migration patterns, human mobility, and rising life expectancy further alter the epidemiological spectrum of the disease [5,6].

Establishing the correct diagnosis is the cornerstone of treatment, and patient care is based on a combination of medical history, ophthalmological examinations, clinical laboratory results, additional imaging studies, and consultations with other specialists. The anatomical location, disease course, and whether one or both eyes are affected simultaneously or sequentially are of primary importance for making a correct diagnosis [7].

The aim of this study was to determine whether the etiology and anatomical location of uveitis influence the course and laterality of the disease, as well as to investigate potential relationships between patient gender and age and the laterality and course of the disease.

## 2. Patients and Methods

By its design, the study falls into the paradigm of observational research. It involved patients with uveitis who were treated at the University Eye Clinic of “St. George” University Hospital in Plovdiv, Bulgaria, over a 13-year period (from January 2011 to December 2023). The research protocol was approved by the ethics committee of the Medical University of Plovdiv, under approval code No. 3, dated 12 March 2025. The study adhered to the guidelines of the 1964 Declaration of Helsinki and its 2000 (Edinburgh) revision. Written informed consent for the publication of data in scientific journals was obtained from the participants.

The participants were hospitalized patients diagnosed with uveitis exhibiting signs of active inflammation, along with those presenting “masquerade syndromes”, where the condition was first categorized as an intraocular inflammatory disorder. For patients with recurrent hospitalizations due to uveitis, the age and duration from the beginning of the first symptoms were regarded as the first illness episode.

Excluded from the study were patients with the following conditions: (1) inflammatory diseases affecting the orbit, ocular adnexa, and ocular coats (such as keratitis and scleritis) that involve the uveal tract; (2) a history of uveitis in which there was no active disease present at the time of the study; and (3) secondary uveitis resulting from trauma, administration of intravitreal medications, or surgical procedures (including cataract extraction, vitrectomy, or glaucoma surgery).

### 2.1. Variables of Research Interest

#### 2.1.1. Anatomical Location

Following the anatomical classification system established by the International Uveitis Study Group (IUSG) [8], uveitis cases were categorized based on the primary anatomical location of the inflammatory process. The categories include anterior uveitis (AU), intermediate uveitis (IU), posterior uveitis (PU), and panuveitis (PAN).

#### 2.1.2. Etiology of Uveitis

All participants underwent an assessment of visual acuity, biomicroscopy, indirect ophthalmoscopy, and tonometry, as well as basic laboratory screening, including a complete blood count, differential blood count, erythrocyte sedimentation rate, creatinine, urea, uric acid, urinalysis, and serological testing for syphilis.

Based on medical examination by a uveitis specialist (M-H.V.), additional ophthalmological tests such as optical coherence tomography, fluorescein angiography, visual field testing, and B-scan ultrasonography were performed to further evaluate the ocular condition. When necessary, some patients also underwent additional tests and consultations with other specialists (rheumatologist, infectious disease specialist, parasitologist, dermatologist, neurologist, pulmonologist, nephrologist, phthisiologist, pediatrician) to confirm or rule out systemic diseases.

Patients were divided into seven groups based on etiology, with the first six groups comprising cases with a clarified cause and the seventh group including all idiopathic cases. This classification was based on the prevalence of etiological factors among our patients to facilitate statistical analysis.

One group included all cases with viral etiology, including herpes simplex virus (HSV), herpes zoster virus (HZV), cytomegalovirus retinitis (CMV), acute retinal necrosis (ARN), Fuchs uveitis syndrome, and Posner–Schlossman syndrome.

The second group consisted of patients with *HLA-B27*-associated uveitis (*HLA-B27*+), including those with acute AU without systemic involvement and those in whom uveitis was associated with a systemic disease, such as ankylosing spondylitis, reactive arthritis, psoriatic arthritis, chronic ulcerative colitis, or Crohn’s disease.

Patients with toxoplasmic retinochoroiditis (TRC), rheumatoid arthritis (RA), and juvenile idiopathic arthritis (JIA) were classified separately. The group of “rare diagnoses” comprised patients with less common diseases observed in our study (one to ten cases). For etiological classification, we applied the following diagnostic criteria: (1) Unilateral, acute, hypertensive, and recurrent AU with sectoral iris paralysis or atrophy and/or a history of herpes keratitis or zoster ophthalmicus in the affected eye, systemic serological tests, and the response to antiviral therapy was considered of herpes viral origin. (2) The *HLA-B27*-associated uveitis group included patients with acute, non-granulomatous, recurrent, and alternating AU who tested positive for the *HLA-B27* antigen. They were also examined by a rheumatologist to confirm the disease based on current rheumatological criteria. (3) Patients with RA and uveitis underwent rheumatological consultation and additional laboratory testing.

In our research, the majority of JIA cases had a diagnosis of arthritis prior to the onset of uveitis. For those in whom uveitis was the first indication of the condition, special serological testing and a consultation with a pediatric rheumatologist were performed.

The diagnosis of toxoplasmic retinochoroiditis (TRC) was based on typical ophthalmoscopic findings, such as fresh yellow-creamy lesions of active retinitis in the posterior pole and associated inflammatory exudation in the vitreous. New lesions often originated from the edge of an atrophic scar with hyperpigmented edges and less frequently from other areas in the fundus. Additionally, these patients underwent specific serological tests, and a positive IgG titer for toxoplasma confirmed the diagnosis.

To lower the cost of gamma-interferon tests, the diagnosis of tuberculous uveitis was conducted in two stages. First, all patients suspected of having tuberculous uveitis underwent a Mantoux skin test, along with a chest X-ray or computed tomography. If the skin test returned positive, patients were then evaluated by a phthisiologist before proceeding to a gamma-interferon test (either QuantiFERON-TB or T-SPOT.TB).

The study also included two patients with HIV infection, both of whom had co-infection with syphilis, two patients with Lyme disease, one with Boutonneuse fever, and one with cat scratch disease. The diagnoses were confirmed through serological testing conducted by an infectious disease specialist. One patient with posterior uveitis caused by toxocariasis was identified based on a positive, specific serological test performed by a parasitologist.

Patients with Behçet’s disease were categorized based on the international criteria for Behçet’s disease [9] and the SUN working group’s classification criteria for uveitis associated with Behçet’s disease [10]. The diagnosis of sarcoid uveitis was validated by biopsy and histological examination. In two instances where patients refused a biopsy, the medical team used the updated diagnostic criteria from the International Workshop on Ocular Sarcoidosis (IWOS) [11] to categorize these patients as having assumed or probable ocular sarcoidosis.

Tubulointerstitial nephritis and uveitis syndrome were diagnosed based on renal biopsy and consultation with a pediatric nephrologist. Multiple sclerosis was confirmed by an expert neurologist using the revised McDonald criteria.

For patients with uveitis linked to systemic lupus, the underlying condition had already been established, whereas those diagnosed with tubulointerstitial nephritis-uveitis syndrome underwent renal biopsy for confirmation. White dot syndrome was diagnosed in four cases based on distinctive ophthalmoscopic characteristics and subsequently confirmed by specialized imaging methods.

Vogt–Koyanagi–Harada (VKH) syndrome was diagnosed in four patients, all of whom were *HLA-DR4*-positive. The patient with sympathetic ophthalmia had a history of perforating trauma to the other eye 15 years earlier, displayed typical clinical signs, and responded well to immunosuppressive therapy. The only case of intraocular lymphoma was confirmed via vitreous biopsy.

Of the cases with masquerade syndrome, two patients were diagnosed with intraocular tumors, one patient with non-Hodgkin’s lymphoma, and five cases with malignancies in other organs, leading to secondary choroidal infiltration.

Patients in whom no associated systemic disease or specific ocular syndrome could be identified were classified as having idiopathic uveitis.

#### 2.1.3. Laterality of Uveitis

Patients were classified as unilateral if only one eye was affected, unilateral alternating if both eyes were involved independently and at different times, and bilateral if both eyes were affected either simultaneously or asynchronously. In unilateral alternating uveitis, either eye can be affected, but only one eye is involved at a time, and the attacks are episodic and recurrent in nature. In bilateral asynchronous disease, the onset may not occur in both eyes at the same time; however, once inflammation begins in the second eye, the first eye remains affected [3].

#### 2.1.4. Number of Affected Eyes

According to the number of affected eyes, patients were divided into two groups: unilateral uveitis—patients with one affected eye—and unilateral alternating and bilateral uveitis—patients with both affected eyes. 

#### 2.1.5. Course of the Disease

Based on the course of uveitis, patients were categorized into three groups, acute, chronic, and recurrent, following the classification established by the SUN Working Group [12]. The acute uveitis group included patients who experienced a sudden onset of the disease, with a limited duration of up to 3 months and no recurrence during the follow-up period. The chronic uveitis group consisted of cases where the inflammatory process reactivated within three months after discontinuing therapy. Recurrent uveitis was characterized by repeated episodes of exacerbation, with remission periods of at least three months without treatment.

#### 2.1.6. Demographic Variables

Patients were categorized by age into six groups: ≤18 years, 19 to 30 years, 31 to 45, 46 to 55 years, 56 to 65 years, and >65 years. Patients’ gender was also a variable of interest. All participants in the study were of Caucasian origin.

### 2.2. Statistical Methods

Data analysis was performed using IBM SPSS Statistics for Windows, Version 27.0 (Armonk, NY, USA: IBM Corp.). Given that the data mostly consisted of categorical and ordinal variables, they are represented as counts and percentages (%). Fisher’s exact test was used for dichotomous variables, whilst the chi-square test and z-test with Bonferroni modifications were utilized for pairwise comparisons across variables with numerous categories (e.g., age groups, anatomical location, and etiology). When anticipated numbers in some cells were below five, the Monte Carlo method was used alongside the chi-square test at a 99% confidence level. Statistical significance was established at *p* < 0.05.

## 3. Results

### 3.1. Demographic and Clinical Data

In this observational study, a total of 934 patients with uveitis were initially included. Of them, 606 met the inclusion criteria, and only their data were used in the analysis. The patients’ ages ranged from 3 to 87 years, with a mean of 46.47 ± 18.56 years. The proportion of patients aged between 31 and 45 years was significantly higher than that of the other five age groups (*p* < 0.05 for all comparisons), whereas patients aged 18 or younger represented the smallest proportion. Males and females constituted similar proportions.

Regarding disease course, acute uveitis and relapse comprised the largest proportions of cases, whereas chronic uveitis was significantly less common.

Anterior uveitis was the most common anatomical location, occurring significantly more often than the other types. The most common types of AU uveitis were idiopathic uveitis (127 cases, 26.7%), ankylosing spondylitis (93 cases, 19.7%), *HLA B27*+ nonsystemic (51 cases, 10.8%), varicella zoster virus uveitis (41 cases, 8.7%), and reactive arthritis (36 cases, 7.6%). Intermediate uveitis was the second most frequent anatomical location, found in 9.6% (*n* = 58) of the cases, with idiopathic uveitis being the most common (*n* = 35, 60.3%). Posterior uveitis came third in frequency with 43 cases (7.1%), with toxoplasmic retinochoroiditis being the most common in this location (*n* = 26, 60.5%). Panuveitis had the least number of cases (33 cases, 5.4%), with the most common types being toxoplasmic retinochoroiditis (7 cases, 21.2%), idiopathic uveitis (5 cases, 15.2%), and masquerade syndromes (5 cases, 15.2%). (See the Supplementary Table S1 for a comprehensive breakdown of uveitis types across anatomical locations).

Unilateral uveitis was significantly more frequent than bilateral. Cases with a clarified etiology were more prevalent than those with an idiopathic etiology (Table 1).

### 3.2. Relationships Among Anatomical Location, Laterality, and Course of Uveitis

Laterality, number of affected eyes, etiology, and disease course all showed significant associations with the anatomical location of uveitis (*p* < 0.001 for all). AU and PU were more frequently unilateral and found in cases with one affected eye. PU had the highest proportion of cases with a clarified etiology. Disease course also varied by anatomical location. The highest proportion of acute inflammation was observed in PU and the lowest in IU. Recurrent uveitis was more frequent in AU and least common in IU. Chronic uveitis was most prevalent in IU and least frequent in AU (Table 2).

### 3.3. Relationship Among Etiology, Laterality, and Course of Uveitis

Laterality and etiology of uveitis showed a significant association (*p* < 0.001). *HLA-B27*-associated uveitis and viral uveitis were more frequently observed in cases with unilateral inflammation. RA-related uveitis and rare etiological diagnoses were more common in patients with bilateral involvement. No significant relationship with laterality was found for toxoplasmosis, JIA, or idiopathic uveitis (Table 3).

A significant association was found between the number of affected eyes and the etiology of uveitis (*p* < 0.001). *HLA-B27*-associated uveitis, RA, and rare diagnoses were more frequently observed in cases involving both eyes—either as unilateral alternating or bilateral involvement. On the other hand, viral uveitis and TRC were more commonly associated with cases affecting only one eye. No significant relationship was found between the number of affected eyes and JIA or idiopathic uveitis (Table 4).

A significant association was observed between the etiology of uveitis and its clinical course (*p* < 0.001). *HLA-B27*-associated uveitis and TRC were more frequently found in recurrent cases. RA, JIA, and rare etiological diagnoses were more prevalent in chronic cases. Viral uveitis did not show a significant association with the course of the disease (Table 5).

A significant association was observed between disease course and the patients’ sex (*p* = 0.021). Men were more frequently associated with acute disease (*p* = 0.009), whereas women more commonly had chronic disease (*p* = 0.042). Laterality and number of affected eyes did not show significant associations with the patients’ sex (Table 6).

Both laterality and the number of affected eyes showed significant associations with the disease course (*p* < 0.001 for both). Unilateral cases were common in acute and recurrent instances, while chronic disease was more frequently observed in cases with bilateral involvement. Acute disease was more prevalent in cases with one affected eye, whereas relapse and chronic disease occurred more often in cases with both eyes affected (Table 7).

The disease course showed a significant association with the patient’s age (*p* = 0.039). Acute disease was most frequently observed in patients aged 19 to 30 years and those over 60 years. Relapse was most prevalent among patients aged 46 to 55 years. Chronic disease was more common in the youngest age cohort (18 years and younger). Laterality and the number of affected eyes did not reveal significant associations with the patient’s age (Table 8).

## 4. Discussion

An important characteristic of uveitis is the involvement of one or both eyes in the inflammatory process, which categorizes it as unilateral, unilateral alternating, bilateral simultaneous, or bilateral asynchronous. In unilateral alternating uveitis, each eye may be affected, but only one eye is involved at a given time, and the attacks are episodic and recurrent. In bilateral asynchronous disease, the onset in both eyes is not simultaneous, but once inflammation begins in the second eye, the first eye continues to be affected. In these cases, the inflammatory process is usually chronic [3]. Following this classification, we investigated whether one or both eyes were affected by uveitis, and in cases where both eyes were involved, whether the uveitis manifested simultaneously or sequentially. Out of all 606 patients in our study, 86.8% had unilateral uveitis, with 97 of them experiencing alternating involvement. Overall, unilateral forms were observed in 70.8%, aligning with other studies that report a higher frequency of unilateral presentation [13,14,15,16,17,18,19].

We observed a significant association between anatomical location and the laterality of uveitis. In our cohort, AU and PU predominantly exhibited unilateral involvement, whereas IU and PAN showed a more balanced distribution between unilateral and bilateral cases. Similar findings have been reported by other researchers [15,20,21]. We attribute these results to the high prevalence of *HLA-B27*-associated uveitis, viral uveitis, and TRC in our study, all of which typically present as unilateral conditions. Among our patients, *HLA-B27*-associated uveitis was the most common, while viral uveitis ranked third.

Both etiologies predominantly presented as AU whereas TRC, the fourth most common cause in our study, manifested as PU. A recent Colombian study by Polania et al. reported similar findings, also noting a higher frequency of unilateral involvement in AU and PU. However, in their study, *HLA-B27*-associated uveitis ranked third in frequency after idiopathic and TRC [20].

An earlier Colombian study by de-la-Torre et al. also found that AU and PU were almost equally unilateral [18]. However, in their cohort, viral uveitis rather than *HLA-B27*-associated uveitis was the third most frequent etiology [18]. Polania et al. attributed this difference to the limited availability of *HLA-B27* genetic testing in the country at the time [20].

A study conducted in Taiwan found that anterior uveitis was predominantly unilateral, with *HLA-B27*-associated uveitis being the most common etiology. Among AU patients, 37% were *HLA-B27* positive [22]. This is a lower proportion compared to Western countries, where approximately 55% of Caucasian patients with acute anterior uveitis are *HLA-B27* positive. For comparison, the carrier rate in the general population is 8–10% [23]. In our study, the prevalence was 41%, closely aligning with reports from Caucasian populations. The slightly lower frequency in our sample can be attributed to the high proportion of idiopathic AU (27%).

*HLA-B27* typing is not covered by health insurance in our country, which may lead to milder cases remaining undiagnosed despite our recommendations. Additionally, the proportion of viral AU among our patients was relatively high (15%). Barisani-Asenbauer et al. reported similar findings, with *HLA-B27*-associated AU identified in 30.5% of patients. In their study, the relative proportions of idiopathic and viral uveitis closely matched ours (42.8% and 13%, respectively). The higher proportion of unclassified AU in their study may be attributed to the earlier study period (1995–2009) when diagnostic methods were not yet widely implemented in clinical practice [24].

Similar findings have been reported in other European studies [25,26,27]. Research from Egypt, India, and Iran showed that approximately half of uveitis cases were bilateral [15,16,28,29,30]. This trend can be explained by the higher prevalence of tuberculosis, sarcoidosis, and Behçet’s disease in Asia and Africa. In a study by Toribio et al. from Spain, bilateral uveitis was also more common (57.8%). The authors attributed this to the increasing immigration to the southern part of the country, where the study was conducted [31]. We believe that the presence of diseases such as VKH, tuberculosis, and syphilis may be responsible for this. In our geographical region, VKH is not a common disease, and although there has been a resurgence of tuberculosis in recent years, the BCG vaccine is included in the mandatory immunization schedule in our country, providing widespread coverage to the population. Our results indicate that bilateral involvement is more frequently seen in cases of uveitis associated with RA and other rare diagnoses.

In our study, the group with rare diagnoses included patients with sarcoidosis, Behçet’s disease, multiple sclerosis, tuberculosis, syphilis, systemic lupus erythematosus, and others, which may explain the observed results. The involvement of one or both eyes is important because some diseases only affect one eye, such as Fuchs’ uveitis syndrome, Posner–Schlossman syndrome, ophthalmomyiasis, and diffuse unilateral subacute neuroretinitis, while others affect both eyes, such as tubulointerstitial nephritis–uveitis syndrome, birdshot chorioretinopathy, and acute multifocal placoid pigment epitheliopathy [32].

The onset of uveitis is critical for its diagnosis. A disease that appears suddenly and has a limited duration is considered acute. In these cases, the body’s response to the damaging agent develops within hours to a few days. Chronic inflammation, on the other hand, has a gradual onset, a persistent course, and a longer duration. It may follow acute inflammation or begin independently [3]. We found that etiology and anatomical location influence the course of uveitis. Posterior uveitis most often has an acute onset (55.8%), followed by AU (48.7%). The latter is also characterized by a recurrent course (45.1%).

A chronic course was typical for patients with IU (65.5%) in our cohort, followed by PAN. Other researchers have also observed an insidious onset, chronic course, and persistent duration for IU [33]. Borde et al. obtained similar results when studying uveitis patterns in a tertiary eye center in central India. They reported an acute onset of the disease in AU and PU, while a chronic course was predominantly observed in IU and PAN [15].

De-la-Torre et al. also found an acute onset in PU (57.9%) and AU (50.9%), with 23.7% of AU cases recurring. The authors reported nearly equal proportions of chronic course in IU (46.3%) and PAN (43.3%) [18]. Based on etiology, we found that TRC and *HLA-B27*-associated uveitis were more frequently recurrent, while uveitis in RA, JIA, and rare diagnoses had a chronic course (*p* < 0.001). Toxoplasmic retinochoroiditis is relatively common in the USA and Europe, accounting for 25–90% of all uveitis cases [20,34,35,36]. The parasite’s affinity for the macula and posterior pole poses a threat to vision, necessitating prevention, early diagnosis, and appropriate treatment.

Throughout a patient’s life, the risk of infection reactivation remains due to the lack of effective treatment for latent toxoplasmic cysts in the retina [37]. According to Jakob et al., the presence of PU, unilateral involvement, and young age are key risk factors for TRC. The authors found that the presence of all three risk factors increases the probability of the disease to 68.8% [25]. Our findings support these observations, as TRC in our study also showed a tendency for recurrence. These results underscore the importance of regular follow-up for affected patients.

The growing use of biologic drugs in therapeutic practice has significantly reduced recurrences of *HLA-B27*-associated uveitis. However, in our cohort, 42% of cases within this etiological group experienced recurrence. We attribute this to the longer time span of our study. In the future, recurrence rates in patients receiving biologic treatment are expected to decline further. Mercanti et al. reported a recurrence rate of 25.49% and recommended monitoring patients at least twice per year after the acute phase [14].

A key characteristic of recurrent acute disease is episodic inflammation, with active phases separated by periods of remission during which the patient is off therapy. In contrast, chronic disease recurs immediately after treatment discontinuation. Recurrent acute disease requires treatment only during active phases, whereas chronic disease necessitates long-term suppressive therapy [3].

The most common form of uveitis in JIA and RA is chronic non-granulomatous AU. In the early stages of the underlying disease, uveitis is often asymptomatic and typically presents with bilateral involvement. Both eyes may be affected simultaneously, or the disease may initially appear unilaterally, with the second eye developing inflammation over several months. This progression highlights the need for frequent and long-term patient follow-up [38,39].

In our study, sex showed a significant association with the course of the disease, with men more frequently experiencing an acute course and women more commonly diagnosed with chronic disease. Various factors may contribute to these differences, including genetics, sex hormones, and social influences [18]. While the exact mechanism remains unclear, it is hypothesized that sex hormones play a role in autoimmune responses, with estrogen enhancing immune activity and androgens exerting an inhibitory effect. Estrogen and androgen receptors are located on the surface of lymphocytes and synovial macrophages, mediating their influence on the immune response [40,41]. According to scientific studies, the effect of estrogen on autoimmunity is dose-dependent, with lower levels being immunostimulatory and higher levels being immunosuppressive [42,43]. Autoimmune diseases are mediated by Th1, Th2, and Th17 cells, with Th1 responsible for cell-mediated immunity and Th2 for humoral immunity. An excessive Th1 immune response leads to tissue damage through the production of IFNγ, while the Th2 response mediates B-cell activation [42,44,45]. Women respond to infection, vaccination, and trauma with a dominant Th2 immune response, leading to increased antibody production, which may explain the chronic course of uveitis. In men, a stronger Th1 immune response is observed, leading to more severe inflammation. This is why the same cause of uveitis manifests with different severity depending on gender [46].

In our study, a significant, although not clearly articulated, association was identified between patients’ age and the course of the disease. Acute disease was most frequently observed in patients aged 19 to 30 and those over 60. Relapse was most prevalent among patients aged 46 to 55, while chronic disease was more common in the youngest group, those aged 18 or younger. These findings suggest that the progression and presentation of the disease may vary for different age groups; however, these age-related differences should be substantiated in further research.

## 5. Limitations

Although it spans an extended period, the current study was conducted at a single tertiary center that treats more complex cases, particularly those that are recurrent or have a chronic course. The results should not be generalized to less complex or acute cases. Furthermore, our diagnostic capabilities were limited because certain tests necessary to determine the etiology are not covered by national health insurance, which forces patients to bear the costs themselves. Because of the limited number of patients with conditions such as sarcoidosis, Behçet’s disease, toxocariasis, syphilis, cat-scratch disease, VKH, SLE, and others, these conditions were grouped into a single category of rare diagnoses. This grouping could potentially obscure the unique characteristics associated with each of these rare conditions.

## 6. Conclusions

Uveitis is a heterogeneous disease, and clinical features such as laterality and disease course, while not entirely definitive, are significant prognostic factors that provide essential clues about the disease’s etiology. Understanding the etiology and progression of the disease helps predict potential outcomes. For instance, chronic uveitis characterized by frequent recurrences can lead to complications, including glaucoma, cataracts, and permanent vision loss. In cases of infectious uveitis, prompt treatment can help prevent the spread of the disease and permanent damage to the body. Awareness of systemic diseases enables comprehensive treatment, thereby reducing the risk of harm to other organs. Evaluating laterality, course, and anatomical localization are crucial characteristics that guide clinicians in achieving faster and more accurate diagnoses, selecting appropriate investigations, administering adequate treatment, improving prognosis, preventing complications, and ensuring effective multidisciplinary care.

Our findings indicate that the etiology and anatomical location of uveitis can serve as prognostic factors for contralateral eye involvement and the progression of the inflammatory process. We found that anterior and posterior uveitis were predominantly unilateral and typically presented with an acute or recurrent course, whereas intermediate uveitis and panuveitis were more commonly chronic. In men, uveitis more often had an acute onset, while in women, it tended to follow a chronic course.

Investigating these relationships is in line with our diagnostic and therapeutic approach to patient management. In certain etiologies, uveitis will predominantly be unilateral, while in others, it will be bilateral with varying degrees of manifestation, necessitating a search for clinical signs in the other eye. In cases where the etiology is unclear, unilateral involvement and the course of the disease will help us make the right decisions, while bilateral involvement will require a different approach.

## Figures and Tables

**Table 1:** Demographic and clinical data.

Variables	Statistics	*p*-Value
Age groups		<0.05 ^z^
o ≤18 years	40 (6.6%) ^a^	
o 19 to 30 years	107 (17.7%) ^b^	
o 31 to 45 years	146 (24.1%) ^c^	
o 46 to 55	97 (16%) ^b^	
o 56 to 65	103 (17%) ^b^	
o >65 years	113 (18.6%) ^b^	
Gender *n* (%)		0.329 ^f^
o Men	314 (51.8%)	
o Women	292 (48.2%)	
Disease course		<0.001 ^z^
o Acute	280 (46.2%) ^a^	
o Relapse	245 (40.4%) ^a^	
o Chronic	81 (13.4%) ^b^	
Anatomic location		<0.001 ^z^
o AU	472 (77.9%) ^а^	
o IU	58 (9.6%) ^b^	
o PU	43 (7.1%) ^b^	
o PAN	33 (5.4%) ^b^	
Laterality		<0.001 ^f^
o Unilateral and unilateral alternating	526 (86.8%)	
o Bilateral—asynchronously and simultaneously	80 (13.2%)	
Number of affected eyes		<0.001 ^f^
o One eye—Unilateral	429 (70.8%)	
o Both eyes—Unilateral alternating and bilateral	177 (29.2%)	
Etiology		<0.001 ^f^
o Clarified	436 (71.9%)	
o Idiopathic	170 (28.1%)	

AU—anterior uveitis; IU—intermediate uveitis; PU—posterior uveitis; PAN—panuveitis; f—Fisher’s exact test; z—z-test. Different superscript letters indicate significant differences at the 0.05 level.

**Table 2:** Relationship among anatomical location and demographic and clinical variables.

Variables	AU	IU	PU	PAN	Chi-Square *p*-Value
Laterality					
o Unilateral	437 (92.6%) ^a^	30 (51.7%) ^b^	36 (83.7%) ^a,c^	23 (69.7%) ^b,c^	
o Bilateral	35 (7.4%)	28 (48.3%)	7 (16.3%)	10 (30.3%)	<0.001
Number of affected eyes					
o One eye	350 (74.2%) ^a^	28 (48.3%) ^b^	31 (72.1%) ^a,b^	20 (60.6%) ^a,b^	
o Both eyes	122 (25.8%)	30 (51.7%)	12 (27.9%)	13 (39.4%)	<0.001
Etiology					
o Clarified	345 (73.1%) ^a^	23 (39.7%) ^b^	40 (93.0%) ^c^	28 (84.8%) ^a,c^	
o Idiopathic	127 (26.9%)	35 (60.3%)	3 (7.0%)	5 (15.2%)	<0.001
Disease course					
o Acute	230 (48.7%) ^a^	13 (22.4%) ^b^	24 (55.8%) ^a^	13 (39.4%) ^a,b^	
o Relapse	213 (45.1%) ^a^	7 (12.1%) ^b^	15 (34.9%) ^a^	10 (30.3%) ^a,b^	
o Chronic	29 (6.1%) ^a^	38 (65.5%) ^b^	4 (9.3%) ^a,c^	10 (30.3%) ^c^	<0.001

AU—anterior uveitis; IU—intermediate uveitis; PU—posterior uveitis; PAN—panuveitis. Different superscript letters indicate significant differences at the 0.05 level.

**Table 3:** Types of etiologies according to laterality.

Etiology	Unilateral and Unilateral Alternating	Bilateral Asynchronous and Simultaneous	Chi-Square Monte-Carlo *p*-Value
o *HLA-B27*+	189 (35.9%) ^a^	8 (10%) ^b^	<0.001
o Viral	98 (18.6%) ^a^	4 (5%) ^b^	
o TRC	36 (6.8%) ^а^	1 (1.3%) ^а^	
o RА	17 (3.2%) ^a^	10 (12.5%) ^b^	
o JIA	14 (2.7%) ^a^	4 (5%) ^a^	
o Rare	30 (5.7%) ^a^	25 (31.3%) ^b^	
o Idiopathic	142 (27%) ^a^	28 (35%) ^a^	

TRC—toxoplasmic retinochoroiditis; RA—rheumatoid arthritis; JIA—juvenile idiopathic arthritis. Different superscript letters indicate significant differences at the 0.05 level.

**Table 4:** Types of etiologies according to the number of affected eyes.

Etiology	One Affected Eye	Two affected Eyes Unilateral Alternating and Bilateral	Chi-Square Monte-Carlo *p*-Value
o *HLA-B27*	125 (29.1%) ^a^	72 (40.7%) ^b^	<0.001
o Viral	96 (22.4%) ^a^	6 (3.4%) ^b^	
o TRC	32 (7.5%) ^а^	5 (2.8%) ^b^	
o RA	13 (3.0%) ^a^	14 (7.9%) ^b^	
o JIA	10 (2.3%) ^a^	8 (4.5%) ^a^	
o Rare	27 (6.3%) ^a^	28 (15.8%) ^b^	
o Idiopathic	126 (29.4%) ^a^	44 (24.9%) ^a^	

TRC—toxoplasmic retinochoroiditis; RA—rheumatoid arthritis; JIA—juvenile idiopathic arthritis. Different superscript letters indicate significant differences at the 0.05 level.

**Table 5:** Types of etiology according to the course of progression.

Etiology *n* (%)	Acute	Relapse	Chronic	Chi-Square *p*-Value
o *HLA-B27*+	94 (33.6%) ^a^	103 (42%) ^b^	0 (0.0%) ^c^	<0.001
o Viral	51 (18.2%) ^a^	35 (14.3%) ^а^	16 (19.8%) ^a^	
o TRC	13 (4.6%) а	23 (9.4%) ^b^	1 (1.2%) ^a^	
o RA	9 (3.2%) ^a^	8 (3.3%) ^a^	10 (12.3%) ^b^	
o JIA	5 (1.8%) ^a^	8(3.3%) ^a,b^	5 (6.2%) ^b^	
o Rare	19 (6.8%) ^a^	14 (5.7%) ^a^	22 (27.2%) ^b^	
o Idiopathic	89 (31.8%) ^a^	54 (22%) ^b^	27 (33.3%) ^a^	

TRC—toxoplasmic retinochoroiditis; RA—rheumatoid arthritis; JIA—juvenile idiopathic arthritis. Different superscript letters indicate significant differences at the 0.05 level.

**Table 6:** Number of eyes and disease course versus patients’ sex.

Variables	Men	Women	Chi-Square *p*-Value
Laterality			
o Unilateral	280 (89.2%) ^a^	246 (84.2%) ^a^	0.073
o Bilateral	34 (10.8%) ^a^	46 (15.8%) ^a^	
Number of affected eyes			
o One eye	223 (71%) ^a^	206 (70.5%) ^a^	0.899
o Both eyes	91 (29%) ^a^	86 (29.5%) ^a^	
Disease course			0.021
o Acute	160 (51%) ^a^	120 (41.1%) ^b^	
o Relapse	121 (38.5%) ^a^	124 (42.5%) ^a^	
o Chronic	33 (10.5%) ^a^	48 (16.4%) ^b^	

Different superscript letters indicate significant differences at the 0.05 level.

**Table 7:** Number of affected eyes versus disease course.

Variables *n* (%)	Acute	Relapse	Chronic	Chi-Square *p*-Value
Laterality				
o Unilateral	259 (92.5%) ^a^	226 (92.2%) ^a^	41 (50.6%) ^b^	<0.001
o Bilateral	21 (7.5%) ^a^	19 (7.8%) ^a^	40 (49.4%) ^b^	
Number of affected eyes				
o One eye	253 (90.4%) ^a^	139 (56.7%) ^b^	37 (45.7%) ^b^	<0.001
o Both eyes	27 (9.6%) ^a^	106 (43.3%) ^b^	44 (54.3%) ^b^	

Different superscript letters indicate significant differences at the 0.05 level.

**Table 8:** Laterality, number of eyes, and disease course versus age groups.

Variables *n* (%)	≤18 Years	19–30 Years	31–45 Years	46–55 Years	56–65 Years	>65 Years	Chi-Square Monte-Carlo *p*-Value
Laterality							
o Unilateral	31	90	123	87	94	101	0.175
	(77.5%)	(84.1%)	(84.2%)	(89.7%)	(91.3%)	(89.4%)	
o Bilateral	9	17	23	10	9	12	
	(22.5%)	(15.9%)	(15.8%)	(10.3%)	(8.7%)	(10.6%)	
Number of affected eyes							
o One eye	28	77	103	62	72	87	0.492
	(70%)	(72%)	(70.5%)	(63.9%)	(69.9%)	(77%)	
o Both eyes	12	30	43	35	31	26	
	(30%)	(28%)	(29.5%)	36.1%)	(30.1%)	(23%)	
Disease course							0.039
o Acute	18 ^a^	61 ^b^	67 ^a^	36 ^a^	39 ^a^	59 ^b^	
	(45%)	(57%)	(45.9%)	(37.1%)	(37.9%)	(52.2%)	
o Relapse	13 ^a^	36 ^a^	61 ^a,b^	50 ^b^	48 ^a,b^	37 ^a,b^	
	(32.5%)	(33.6%)	(41.8%)	(51.5%)	(46.6%)	(32.7%)	
o Chronic	9 ^a^	10 ^b^	18 ^a,b^	11 ^a,b^	16 ^a,b^	17 ^a,b^	
	(22.5%)	(9.3%)	(12.3%)	(11.3%)	(15.5%)	(15%)	

Different superscript letters indicate significant differences at the 0.05 level.

## Data Availability

The corresponding author is available for requests regarding access to the data.

## Supplementary Materials

The following supporting information can be downloaded at: https://www.mdpi.com/article/10.3390/life15060882/s1, Table S1: Distribution of uveitis according to etiological diagnosis and anatomical localization of uveitis.
