# Supplementary material for: Etiology and Anatomical Location of Uveitis—Prognostic Factors for Disease Course and Laterality
Source: Life (Basel). 2025 May 30;15(6):882. doi: 10.3390/life15060882 (PMC12194477; doi:10.3390/life15060882)
Supplement: Supplementary file 1 [file life-15-00882-s001.zip › life-3616311-supplementary.pdf]

Supplementary table: Distribution of uveitis according to etiological diagnosis and anatomical localization of uveitis

**Etiology**	**Anatomic location**				**Total**
	AU	IU	PU	PAN	
Ankylosing spondylitis	93	1	0	0	94
Crohn's disease	3	0	0	0	3
Intraocular lymphoma	0	1	0	0	1
Idiopathic uveitis	127	35	3	5	170
Lyme disease	0	2	0	0	2
Boutonneuse fever	0	0	1	0	1
Masquerade syndromes	2	1	0	5	8
Multiple sclerosis	0	4	0	0	4
Sympathetic ophthalmia	0	0	0	1	1
Posner-Schlossman syndrome	13	0	0	0	13
Psoriatic arthritis	10	1	0	0	11
Rheumatoid arthritis	24	2	0	1	27
Sarcoidosis	1	1	1	2	5
Behçet's disease	3	0	1	3	7
Cat scratch disease	0	1	0	0	1
Reactive arthritis	36	0	0	0	36
Syphilis	1	0	0	0	1
Syphilis_HIV	1	1	0	0	2
SLE	0	2	0	0	2
Tuberculosis	7	0	1	0	8
TINU	3	0	0	0	3
Toxocariasis	0	0	1	0	1
TRC	0	4	26	7	37
Fuchs uveitis syndrome	6	0	0	0	6
HZV	41	0	0	0	41
HSV	31	1	3	1	36
UC	2	0	0	0	2
JIA	16	1	0	1	18
ARN	0	0	0	3	3
CMV	0	0	0	3	3
HLA B27+ nonsystemic	51	0	0	0	51
VKH	1	0	2	1	4
White dot Sy	0	0	4	0	4
**Total**	472	58	43	33	606

JIA – juvenile rheumatoid arthritis; HSV – herpes simplex virus uveitis; HZV – varicella zoster virus uveitis; SLE – systemic lupus erythematosus; CMV – cytomegalovirus uveitis; TB – tuberculosis; TINU – tubulointerstitial nephritis and uveitis; UC – ulcerative colitis; ARN – acute retinal necrosis; VKH – Vogt-Koyanagi-Harada syndrome; TRC – toxoplasmic retinochoroiditis; AU – anterior uveitis; IU – intermediate uveitis; PU – posterior uveitis; PAN – panuveitis
